# Impact of atrial fibrillation on outcomes in asymptomatic severe aortic stenosis: a propensity-matched analysis

**DOI:** 10.3389/fcvm.2023.1195123

**Published:** 2023-06-20

**Authors:** Didem Oguz, Geoffrey D. Huntley, Edward A. El-Am, Christopher G. Scott, Jeremy J. Thaden, Sorin V. Pislaru, Katarina L. Fabre, Mandeep Singh, Kevin L. Greason, Juan A. Crestanello, Patricia A. Pellikka, Jae K. Oh, Vuyisile T. Nkomo

**Affiliations:** ^1^ Department of Cardiovascular Medicine, Mayo Clinic, Rochester, MN, United States; ^2^ Biomedical Statistics and Informatics, Mayo Clinic, Rochester, MN, United States; ^3^ Department of Internal Medicine, Mayo Clinic, Rochester, MN, United States; ^4^ Department of Cardiovascular Surgery, Mayo Clinic, Rochester, MN, United States

**Keywords:** atrial fibrillation, asymptomatic, aortic stenosis, right ventricle dysfunction, mitral regurgitation

## Abstract

**Background:**

Atrial fibrillation (AF) portends poor prognosis in patients with aortic stenosis (AS).

**Objectives:**

This study aimed to study the association of AF vs. sinus rhythm (SR) with outcomes in asymptomatic severe AS during routine clinical practice.

**Methods:**

We identified 909 asymptomatic patients from 3,208 consecutive patients with aortic valve area ≤1.0 cm^2^ and left ventricular ejection fraction ≥50% at a tertiary academic center. Patients were grouped by rhythm at the time of transthoracic echocardiogram [SR: 820/909 (90%) and AF: 89/909 (10%)]. Propensity-matched analyses (2 SR:1 AF) matching 174 SR to 89 AF patients by age, sex, and clinical comorbidities were used to compare outcomes.

**Results:**

In the propensity-matched cohort, median age (82 ± 8 vs. 81 ± 9 years, *p* = 0.31), sex distribution (male 58% vs. 52%, *p* = 0.30), and Charlson comorbidity index (4.0 vs. 3.0, *p* = 0.26) were not different in AF vs. SR. Median follow-up duration was 2.6 (IQR: 1.0–4.4) years. The 1-year rate of aortic valve replacement (AVR) was not different (AF: 32% vs. SR: 37%, *p* = 0.31). All-cause mortality was higher in AF [hazard ratio (HR): 1.68 (1.13–2.50), *p* = 0.009]. Independent predictors of mortality were age [HR: 1.92 (1.40–2.62), *p* < 0.001], Charlson comorbidity index [1.09 (1.03–1.15), *p* = 0.002], aortic valve peak velocity [HR: 1.87 (1.20–2.94), *p* = 0.006], stroke volume index [HR: 0.75 (0.60–0.93), *p* = 0.01], moderate or more mitral regurgitation [HR: 2.97 (1.43–6.19), *p* = 0.004], right ventricular systolic dysfunction [HR: 2.39 (1.29–4.43), *p* = 0.006], and time-dependent AVR [HR: 0.36 (0.19–0.65), *p* = 0.0008]. There was no significant interaction of AVR and rhythm (*p* = 0.57).

**Conclusions:**

Lower forward flow, right ventricular systolic dysfunction, and mitral regurgitation identified increased risk of subsequent mortality in asymptomatic patients with AF and AS. Additional studies of risk stratification of asymptomatic AS in AF vs. SR are needed.

## Introduction

The natural history of patients with asymptomatic severe aortic stenosis (AS) is well established (1–5). About half of patients are asymptomatic at the time of severe AS diagnosis (3, 6), and approximately 19% of patients are asymptomatic at the time of referral for treatment of severe AS (7). Class I indications for aortic valve replacement (AVR) in the setting of asymptomatic severe AS are left ventricular ejection fraction (LVEF) <50% or if undergoing open heart surgery for other reasons (8, 9). However, some patients with asymptomatic severe AS and preserved LVEF ≥50% are at increased risk of mortality and there is growing evidence to support consideration of early AVR in those with high-risk features, for example, very severe AS (transaortic valve peak velocity >5 m/s) (Class 2a indication).

Atrial fibrillation (AF) is a common cardiac arrhythmia that can occur without symptoms (10). AF is frequently encountered in patients with AS (11, 12) and when present is associated with higher morbidity and mortality (13–17). Among asymptomatic patients with preserved LVEF, AF was present in 23% of patients with moderate–severe AS (14), in 15% of patients with severe AS (15), and in 6% of patients with very severe AS in the Randomized Comparison of Early Surgery vs. Conventional Treatment in Very Severe Aortic Stenosis (18).

A previous study showed excess mortality in asymptomatic patients with AF and moderate-severe AS and preserved LVEF, without a difference in overall mortality under medical management or medical and surgical management (14). The aim of this study was to examine clinical outcomes, including rates of AVR and determinants of overall mortality, in patients with asymptomatic severe AS with preserved LVEF ≥50% and coexistent AF vs. sinus rhythm (SR). We hypothesized that AF compared to SR is associated with increased risk of mortality in patients with asymptomatic severe AS and preserved LVEF and that AVR improves outcomes.

## Methods

### Study population

A cohort of consecutive patients aged ≥18 years who had aortic valve area (AVA) ≤1 cm^2^ or AVA indexed ≤0.6 cm^2^/m^2^ and LVEF ≥50% by transthoracic echocardiography (TTE) at Mayo Clinic, Rochester, MN, United States, from January 1, 2008, to December 31, 2016, was identified retrospectively from the Mayo Clinic Echocardiographic Laboratory Database. Patients were divided by rhythm (AF vs. SR) at the time of TTE. Patients with concomitant moderate or severe aortic regurgitation, prosthetic aortic valve, subvalvular or supravalvular AS, dynamic subaortic obstruction, or active endocarditis were excluded. Patients in sinus rhythm during the TTE but with a history of paroxysmal AF were considered in the SR cohort. Information on symptom status at the time of index TTE was obtained from review of the electronic medical record. If the patient did not have any record of dyspnea, syncope, or angina symptoms attributed to AS in proximity to the index TTE showing severe AS, we classified the case as asymptomatic severe AS. Asymptomatic patients who had a positive exercise stress test were classified as symptomatic and excluded. Relationship of symptom status as extracted from the medical record and subsequent observed overall survival is shown in Supplementary Figure S1 and the asymptomatic group reassuringly had better overall survival compared to the symptomatic group.

Clinical data including medical history, electrocardiogram, and serum blood chemistries were obtained from the electronic medical records. Hypertension was defined as systolic blood pressure ≥140 mmHg, diastolic blood pressure ≥90 mmHg, history of hypertension, or reported current use of antihypertensive medication. Diabetes mellitus was defined as fasting blood sugar >126 mg/dl on two occasions or treatment with antidiabetic agents. Renal insufficiency was defined as serum creatinine ≥1.3 mg/dl. Chronic lung diseases included obstructive or restrictive lung disease. Charlson comorbidity index (CCI) was calculated (19). Outcome data included subsequent AVR (surgical or transcatheter), cardiovascular mortality, and all-cause mortality. Vital status was at the latest follow-up visit at Mayo Clinic and was determined from the medical records and Minnesota death certificates. Only individuals who had granted permission for use of their medical records for research (according to the Minnesota Research Authorization) were included. The Mayo Clinic Institutional Review Board approved the study.

### Echocardiographic data

Echocardiographic studies were performed in accordance with the American Society of Echocardiography/European Association of Echocardiography (ASE/EAE) guidelines (20, 21) and stored digitally. Echocardiographic data reported were from the comprehensive TTE studies performed during routine clinical practice. Left ventricular outflow tract (LVOT) diameter was measured from the parasternal long-axis view in early-systole at the tissue–blood interface insertion points of the aortic valve cusps. Forward stroke volume was determined as the product of the LVOT area and LVOT pulsed-wave time velocity integral (TVI). AVA was calculated by the continuity equation as the forward Doppler stroke volume divided by the continuous-wave Doppler TVI signal across the aortic valve. Averages of three and five consecutive Doppler signals were used for patients in SR and in AF, respectively. Severe AS was defined as AVA ≤1.0 cm^2^ or AVA indexed to body surface area ≤0.6 cm^2^/m^2^ (20); high-gradient severe AS (HGAS) was defined as aortic valve peak velocity ≥4 m/s or mean gradient (MG) ≥40 mmHg, and low-gradient severe AS (LGAS) was defined as aortic valve peak velocity <4 m/s and MG <40 mmHg (8). Quantitative Doppler was used over qualitative parameters for grading severity of valvular regurgitation (20). Etiology of atrioventricular valvular regurgitation was classified as organic vs. functional vs. mixed (functional and organic) (22). Cardiac chamber size and function were determined according to the guidelines and biplane Simpson's method was used for calculation of LVEF (21). Right ventricle (RV) size and function were based on quantitative, where available, and semiquantitative assessment, based on current guidelines (21, 23). The size of the RV was assessed semiquantitatively and compared to the size of the left ventricle (LV). The RV was considered normal if it was two-thirds or less in size compared to the LV. The RV was at least mildly enlarged if it was the same size as the LV and at least moderately enlarged if it was larger than the LV and occupied the apex. Quantitative assessment of RV size was estimated from RV longitudinal diameter, RV mid cavity diameter, and RV basal diameter measured from a four-chamber view obtained from the apical window focused on the RV at end-diastole. Classification of RV dysfunction was based on semiquantitative assessment, which integrated the visual assessment of the contractility of the RV free wall, apex, outflow tract, and interventricular septum from multiple views including the parasternal long-axis, short-axis, apical four-chamber, apical long- and short-axis, and subcostal views, and integrated visual assessment of the displacement of the tricuspid annulus (21). Quantitative assessment of RV function [systolic excursion velocity (*S*′) or tricuspid annular plane systolic excursion (TAPSE)] was not available for all patients. Semiquantitative assessment of RV dysfunction has previously shown to have very good reliability and consistency (24) and, where available, correlated well with *S*′ and TAPSE. RV systolic pressure was estimated using tricuspid regurgitation velocity plus estimated right atrium pressure.

### Statistical analysis

Categorical variables were expressed as numbers and percentages, and continuous variables were reported as mean ± SD or median and interquartile range. Continuous variables were compared across groups using Student’s *t*-test or Wilcoxon's rank-sum test, as appropriate. Categorical variables were compared using *χ*^2^ or Fisher's exact test. Propensity matching was used to match each AF patient with up to two SR patients. Variables included in the propensity matching were demographics, clinical comorbidities, and year of echocardiogram. Rates of overall mortality and AVR were estimated by Kaplan–Meier methods, and groups were compared using the log-rank test. The cumulative incidence of cardiac death was estimated accounting for the competing risk of noncardiac death and groups were compared with Gray's test. Cox proportional hazards regression was used to examine associations with risk of mortality and rates of AVR. AVR was evaluated as a time-dependent covariate for overall mortality. An interaction term was included in the model to test for differential benefit of AVR by rhythm. Two different multivariable models were created, one using only clinical variables and another using clinical and echocardiographic variables. Clinical adjustment variables were chosen *a priori* based on their known prognostic association. Echocardiographic variables were chosen using backward elimination from those that were more than 80% complete and were significantly different between groups. These variables were individually removed until the model only contained variables with *p* < 0.05. When selecting the echocardiographic variables, missing values were imputed with the median value and a missing indicator was included in the model for adjustment. A second Kaplan–Meier analysis of mortality was performed after censoring at AVR to examine outcomes under medical therapy. SAS version 9.4 (Cary, NC, United States) was used for analyses, and two-sided *p*-values <0.05 were considered statistically significant.

## Results

A total of 909/3,208 patients (28%) met the inclusion criteria of asymptomatic severe AS and preserved LVEF ≥50%. The mean age was 76 ± 11 years, and 423 patients (47%) were female. SR was present in 820/909 (90%) and AF in 89/909 (10%) during the index TTE; the patients in AF were older (82 ± 8 vs. 75 ± 11 years, *p* < 0.001). Baseline characteristics of the overall cohort are shown in Supplementary Table S1.

Propensity matching was of the 89 patients in AF to 174 patients in SR. The remaining results and analyses presented below are of the propensity-matched cohort.

### Propensity-matched cohort

#### Baseline characteristics

Baseline clinical characteristics of the propensity-matched cohort are shown in Table 1. The mean age was 82 ± 8 vs. 81 ± 9 years (*p* = 0.31), and 58% vs. 52% were male (*p* = 0.30) in patients in AF and SR, respectively. There were no differences in clinical characteristics, except serum levels of N-Terminal Pro-B-Type Natriuretic Peptide (NT-pro-BNP) were higher in AF compared to SR [1,916 pg/ml (IQR: 1,029–3,117) vs. 775 pg/ml (IQR: 257–2,057), *p* < 0.001].

**Table 1:** Characteristics of propensity-matched cohort of asymptomatic patients with severe aortic stenosis.

Variable	SR (*N* = 174)	AF (*N* = 89)	*p*-value
Age	81 ± 9	82 ± 8	0.31
Male gender, *n* (%)	90 (52%)	52 (58%)	0.30
Body mass index	29.0 ± 6.4	27.9 ± 5.3	0.17
Hypertension, *n* (%)	135 (78%)	64 (72%)	0.31
Diabetes mellitus, *n* (%)	68 (39%)	30 (34%)	0.88
Congestive heart failure, *n* (%)	68 (39%)	34 (38%)	0.89
Chronic obstructive pulmonary disease, *n* (%)	11 (6%)	8 (9%)	0.43
Stroke, *n* (%)	55 (32%)	26 (29%)	0.69
Renal failure, *n* (%)	52 (30%)	25 (28%)	0.76
Hyperlipidemia, *n* (%)	118 (68%)	60 (67%)	0.95
Coronary artery disease, *n* (%)	97 (56%)	50 (56%)	0.95
PCI history, *n* (%)	23 (13%)	10 (11%)	0.65
CABG history, *n* (%)	25 (14%)	15 (17%)	0.60
Charlson comorbidity index, median (Q1, Q3)	3.0 (1.0, 6.0)	4.0 (2.0, 7.0)	0.26
Anticoagulation, *n* (%)	13 (13%)	67 (75%)	<.001
Creatinine, median (Q1, Q3)	1.1 (0.9, 1.4)	1.1 (0.9, 1.3)	0.41
Hemoglobin, median (Q1, Q3)	12.6 (11.1, 13.6)	12.6 (11.3, 13.9)	0.49
NT-pro-BNP, median (Q1, Q3)	775 (257, 2,057)	1,916 (1,029, 3,117)	<.001
Ejection fraction, %	65 ± 6	62 ± 6	0.001
Mean gradient, mmHg	43 ± 12	40 ± 15	0.10
Peak velocity, m/s	4.2 ± 0.6	4.0 ± 0.7	0.035
Aortic valve area, cm^2^	0.9 ± 0.1	0.8 ± 0.1	0.11
Aortic valve area index, cm^2^/m^2^	0.6 ± 0.1	0.5 ± 0.1	0.09
Low-gradient AS, *n* (%)	52 (30%)	39 (44%)	0.025
LV end diastolic diameter, mm	47 ± 5	47 ± 5	0.77
LV end systolic diameter, mm	29 ± 4	30 ± 5	0.06
Stroke volume index, ml/m^2^	52 ± 11	44 ± 10	<.001
LV mass index, g/m^2^	110 ± 27	107 ± 29	0.42
Right ventricular systolic pressure, mmHg	37 ± 12	45 ± 14	<.001
TAPSE, mm	22 ± 5	16 ± 4	<.001
Left atrial volume index, ml/m^2^	43 ± 14	59 ± 15	<.001
*E*/*e*′	18 ± 8	20 ± 10	0.26
*Q*, ml/s	260 ± 42	242 ± 44	0.001
Abnormal right ventricle, *n* (%)	7 (4%)	22 (26%)	<.001
Mitral regurgitation^a^, *n* (%)	6 (3%)	6 (7%)	0.20
Tricuspid regurgitation^a^, *n* (%)	8 (5%)	19 (22%)	<.001
Mitral or tricuspid regurgitation^a^, *n* (%)	12 (7%)	21 (25%)	<.001

SR, normal sinus rhythm; AF, atrial fibrillation; PCI, percutaneous coronary intervention; CABG, coronary artery bypass graft; LV, left ventricle; NT-pro-BNP, N-terminal Pro-B-Type Natriuretic Peptide; *Q*, transaortic flow rate; TAPSE, tricuspid annular plane systolic excursion; *E*, early mitral inflow Doppler velocity; *e*′, mitral annulus tissue Doppler velocity.

^a^ Moderate or greater in severity.

Baseline echocardiographic characteristics of the propensity-matched cohort are also shown in Table 1. The mean LVEF was 62 ± 6% in AF and 65 ± 6% in SR (*p* < 0.001). Patients in AF had a lower stroke volume index (44 ± 10 vs. 52 ± 11 ml/m^2^, *p* < 0.001), more prevalent low-gradient AS (44% vs. 39%, *p* = 0.025), larger left atrial volume index (59 ± 15 vs. 43 ± 14 ml/m^2^, *p* < 0.001), higher right ventricular systolic pressure (45 ± 14 vs. 37 ± 12 mmHg, *p* < 0.001), more prevalent right ventricular systolic dysfunction (26% vs. 4%, *p* < 0.001), and more prevalent moderate or more mitral or tricuspid regurgitation (25% vs. 7%, *p* < 0.001) compared to patients in SR. The etiology of mitral regurgitation in patients with AF was functional in 83% and mixed (functional and organic) in 17%, while the etiology in patients with SR was organic in 50%, functional in 33%, and mixed in 17%. None of the patients in AF or SR with mitral regurgitation had mitral valve prolapse or flail.

### Outcomes

**Figure 1:**
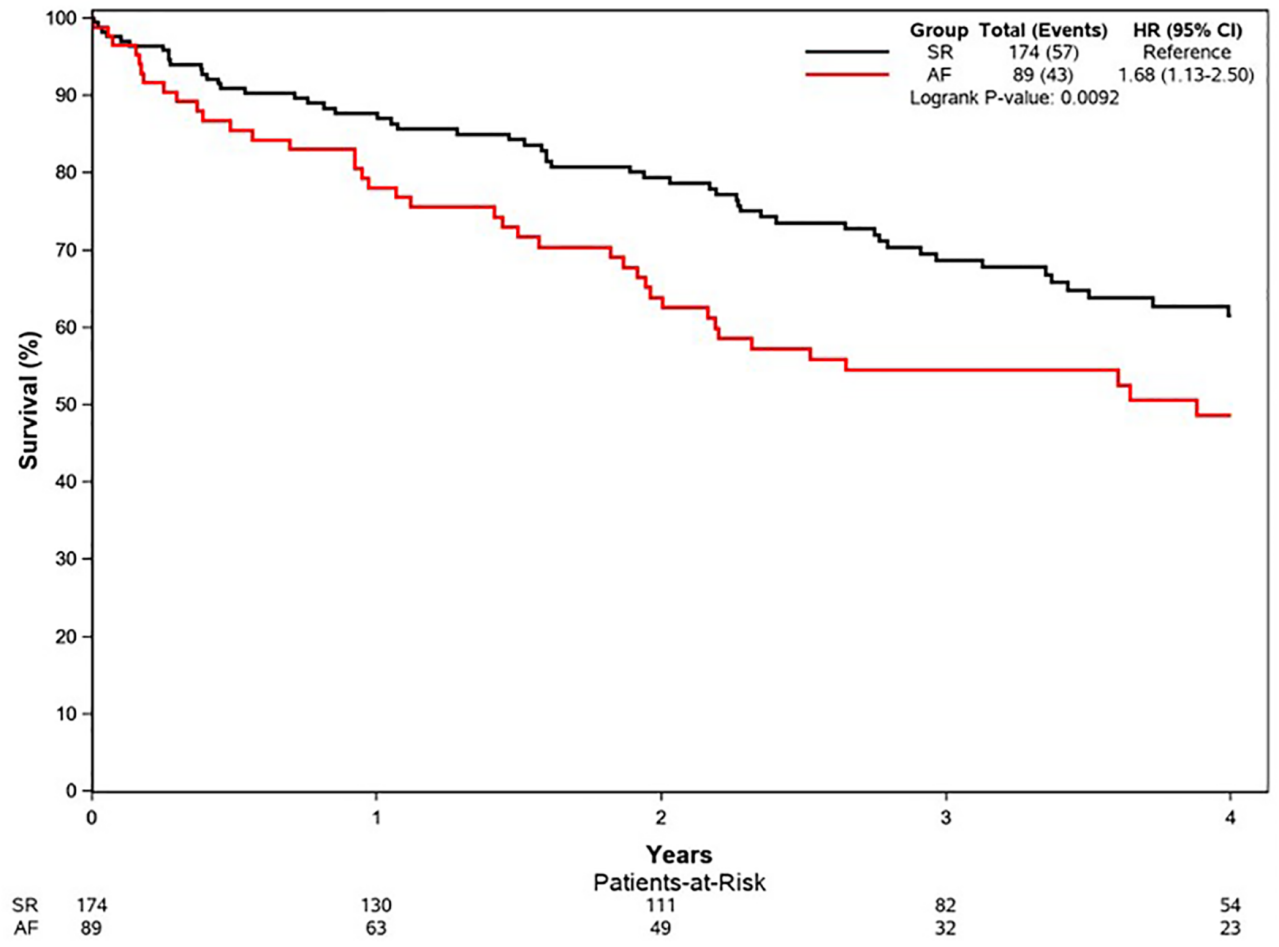
Overall survival in the propensity-matched cohort. Kaplan–Meier curves for overall survival in each group. Survival was worse in the AF group vs. the SR group (*p* = 0.009). AF, atrial fibrillation; SR, normal sinus rhythm.

**Figure 2:**
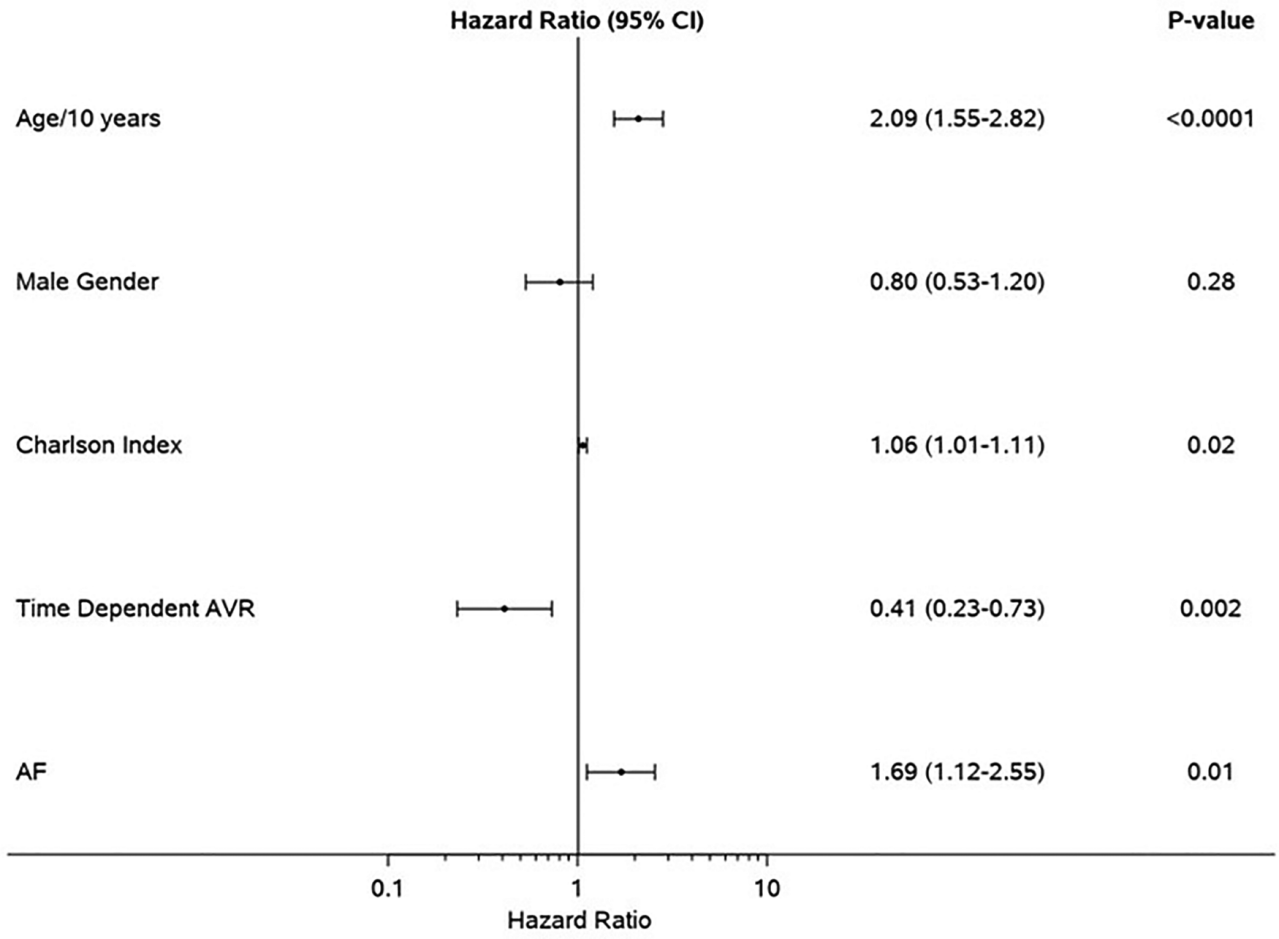
Clinical determinants of mortality in the propensity-matched cohort. Forest plot of multivariable clinical predictors of mortality. Hazard ratios, 95% CIs, and *p*-values from multivariable analyses are shown. AVR, aortic valve replacement; AF, atrial fibrillation.

**Figure 3:**
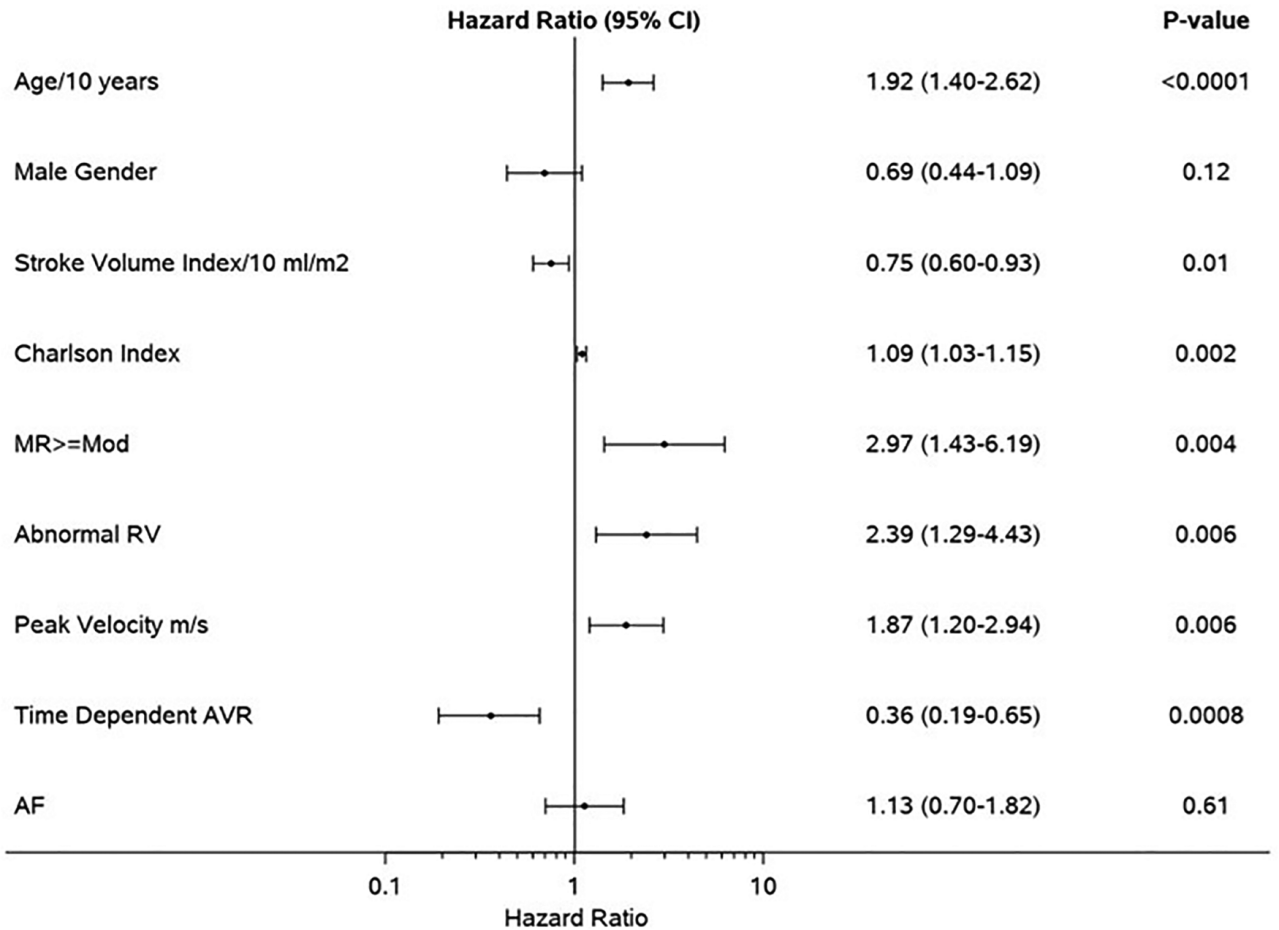
Clinical and echocardiographic determinants of mortality in the propensity-matched cohort. Forest plot of multivariable predictors of mortality. Hazard ratios, 95% CIs, and *p*-values from multivariable analyses are shown. MR, moderate or greater mitral regurgitation; RV, right ventricle; AVR, aortic valve replacement; AF, atrial fibrillation.

Median follow-up duration was 2.6 (IQR: 1.0–4.4) years. Follow-up AVR was performed in 87 patients in the SR group and 36 patients in AF group, and the 1-year rate of AVR was not different in AF (32%) vs. SR (37%), *p* = 0.31. Development of symptoms was the indication for AVR in 97/123 of the patients (79%) (SR 83% vs. AF 69%, *p* = 0.10). The type of aortic valve replacement (surgical vs. transcatheter) was not different in AF (surgical 70%) vs. SR (surgical 64%), *p* = 0.58. During follow-up, 57 patients in SR and 43 patients in AF died; the AF group had higher all-cause mortality compared to SR group [hazard ratio (HR): 1.68 (1.13–2.50) *p* = 0.009]. The 1-year survival rate was higher in the SR group (88%) (Figure 1). AVR was associated with significant reduction in risk of death [HR: 0.41 (0.23–0.73), *p* = 0.002], and there was no interaction of AVR and rhythm (*p* = 0.57). Rates of AVR within 1 year were higher in HGAS compared to LGAS in both SR (48% vs. 11%, *p* < 0.001) and AF (41% vs. 19%, *p* = 0.04). Independent clinical determinants of mortality were age [HR/10 years 2.09 (1.55–2.82), *p* < 0.0001], Charlson comorbidity index [HR: 1.06 (1.01–1.11), *p* = 0.02], time-dependent AVR [HR: 0.41 (0.23–0.73, *p* = 0.002], and AF [HR: 1.69 (1.12–2.55), *p* = 0.01] (Figure 2). However, when factoring in echocardiographic findings (Figure 3), age [HR: 1.92 (1.40–2.62), *p* < 0.001], Charlson comorbidity index [1.09 (1.03–1.15, *p* = 0.002], and time-dependent AVR [HR: 0.36 (0.19–0.65), *p* = 0.0008] remained independent determinants of mortality, and stroke volume index [HR: 0.75 (0.60–0.93), *p* = 0.01], right ventricular systolic dysfunction [HR: 2.39 (1.29–4.43), *p* = 0.006], moderate or more mitral regurgitation [HR: 2.97 (1.43–6.19, *p* = 0.004], and aortic valve peak velocity [HR: 1.87 (1.20–2.94), *p* = 0.006] were independent echocardiographic determinants of mortality; AF was no longer an independent predictor of mortality after adjusting for factors closely associated with AF (Figure 3).

## Discussion

This study of patients with asymptomatic severe AS and preserved LVEF compared outcomes in those in SR vs. AF at the time of transthoracic echocardiogram diagnosis of severe AS. The main findings were: (1) AF was not infrequent in patients with asymptomatic severe AS; (2) overall mortality was worse in patients with asymptomatic severe AS and concomitant AF compared to a propensity-matched cohort in SR despite similar rates of AVR; (3) lower forward flow, right ventricular systolic dysfunction, and moderate or more mitral valve regurgitation identified higher risk of subsequent mortality in asymptomatic patients with AF and AS; and (4) AVR was associated with improved outcomes irrespective of rhythm.

The natural history of asymptomatic AS is well described and overall survival when left ventricular systolic function is preserved is good even without AVR (3). However, while outcomes are favorable, recent efforts have been made to identify features associated with a high risk of developing symptoms, left ventricular dysfunction, or AS-related events to improve outcomes both with medical management and AVR. For example, asymptomatic patients with peak aortic valve velocities >5.0 m/s (very severe AS) have a high rate of symptom onset and a substantially increased risk of cardiovascular mortality (4, 25). In addition, patients with rapid hemodynamic progression (peak velocity progression >0.3 m/s/year) and significant valve calcification have a high rate of symptom development and mortality (2). Progressive decline in LVEF <60% on serial studies is another high-risk feature predictive of worse outcomes (26–28). Contemporary guidelines on the management of patients with valve disease acknowledge this growing evidence and consider surgical AVR reasonable in asymptomatic patients with these high-risk features (8).

The association of worse prognosis in patients with severe AS and concomitant AF compared to SR is well documented in the literature (15, 29). This increased risk is in part related to older age, higher clinical comorbidity burden, and lower referral rates to AVR in patients with AF (14, 15, 29, 30). We matched the patients with AF and SR by age, sex, and clinical comorbidities to remove the effect of these differences on outcomes. Indeed, after propensity matching by age, sex, and clinical comorbidities, there was no difference in rates of AVR between patients in AF vs. SR, in contrast to unmatched cohorts showing lower rates of AVR among patients with AF (15, 31). However, AF was still associated with increased risk of mortality even in the propensity-matched cohort, and the higher mortality in patients with AF was linked to lower forward flow, and to more prevalent right ventricular systolic dysfunction and mitral regurgitation in patients with AF. Varying degrees of structural changes of atrial enlargement, annulus dilatation with atrioventricular valvular regurgitation, increased pulmonary pressures, and right ventricular systolic dysfunction are more common in patients with AF and occur even in the absence of AS (32–35). This explains the differences in the baseline echocardiographic characteristics in the propensity-matched cohort, which were reflective of the adverse hemodynamic sequelae of AF (15, 34–36), resulting in more pronounced extent of cardiac damage in the AF group compared to the SR group (37).

The most common indication for AVR in this cohort was the development of new symptoms, consistent with previously published observations (3, 4). However, symptoms from AS can be very similar to symptoms from AF, and patients with AF and symptoms in the setting of severe AS are sometimes not referred for AVR because clinicians attribute symptoms to AF instead of AS (15). In addition, it is known that patients with low-gradient compared to high-gradient AS have lower rates of AVR (31, 38, 39), and the lower rates of AVR in low-gradient AS have been shown to be more detrimental to patients with AF (31). Since AVR was associated with improvement in overall survival in both the AF and SR groups, the findings of the current study generate important clinical hypotheses about risk stratification in asymptomatic AS and whether the presence of AF should be considered a marker of increased risk of mortality and prompt referral to AVR.

### Study limitations

This is a single center retrospective study. The duration of AF could not be determined precisely, although patients in AF during the echocardiogram typically have chronic persistent AF (15, 31). Baseline symptom status was based on clinical assessment and what was documented in the medical record around the time of diagnosis of severe AS. Systematic exercise testing to determine true symptom status was not performed. However, asymptomatic patients that did undergo exercise stress testing based on clinical indication were excluded if they had a positive stress test. Importantly, symptom status extracted from the medical record was able to discriminate between those at higher vs. lower risk of mortality during follow-up, irrespective of rhythm (Supplementary Figure S1), which means those that were determined to be asymptomatic during routine clinical practice had a better outcome than those diagnosed as symptomatic. Baseline NT pro-BNP levels were not checked in 38% of the propensity-matched cohort. However, the lack of NT pro-BNP levels was likely reflective of the lack of clinical indication for measurement of serum NT pro-BNP levels.

## Conclusions

Among propensity-matched asymptomatic patients with severe AS and preserved left ventricular ejection fraction, concomitant AF vs. SR was associated with excess mortality and linked to lower forward flow, right ventricular systolic dysfunction, and mitral regurgitation. Aortic valve replacement was associated with improved outcomes irrespective of rhythm. Further studies are needed to examine appropriate risk stratification in patients with AS and concomitant AF.

## Data Availability

The raw data supporting the conclusions of this article will be made available by the authors, without undue reservation.

## Supplementary material

Supplementary Figure 1 Overall survival based on symptoms status at time of diagnosis of severe aortic stenosis. Kaplan-Meier curves for overall survival in each group based on baseline symptoms status. Survival was worse in the symptomatic versus asymptomatic group, irrespective of rhythm. Normal sinus rhythm (Panel A; *p* < 0.001) and atrial fibrillation (Panel B; *p* = 0.06). Adjusted for age, sex, and Charlson Comorbidity Index. Abbreviations: AF, atrial fibrillation; SR, normal sinus rhythm.

Supplementary Figure 2 Observed survival in overall cohort. Kaplan-Meier curves for overall survival in the overall cohort based on rhythm. Survival was worse for patients in AF versus SR (*p* < 0.001). Abbreviations: AF, atrial fibrillation; SR, normal sinus rhythm.

Supplementary Figure 3 Clinical determinants of mortality in the overall cohort. Forest plot of multivariable clinical predictors of mortality. Hazard ratios, 95% confidence intervals, and *p*-values from multivariable analyses are shown. Abbreviations: AVR, aortic valve replacement; AF, atrial fibrillation.

Supplementary Figure 4 Clinical and echocardiographic determinants of mortality in the overall cohort. Forest plot of multivariable clinical predictors of mortality. Hazard ratios, 95% confidence intervals, and *p*-values from multivariable analyses are shown. Abbreviations: RV, right ventricle; PASP, pulmonary artery systolic pressure; AVR, aortic valve replacement; AF, atrial fibrillation.
