# Supplementary figures and images for: Impact of atrial fibrillation on outcomes in asymptomatic severe aortic stenosis: a propensity-matched analysis
Source: Front Cardiovasc Med. 2023 Jun 20;10:1195123. doi: 10.3389/fcvm.2023.1195123 (PMC10318187; doi:10.3389/fcvm.2023.1195123)

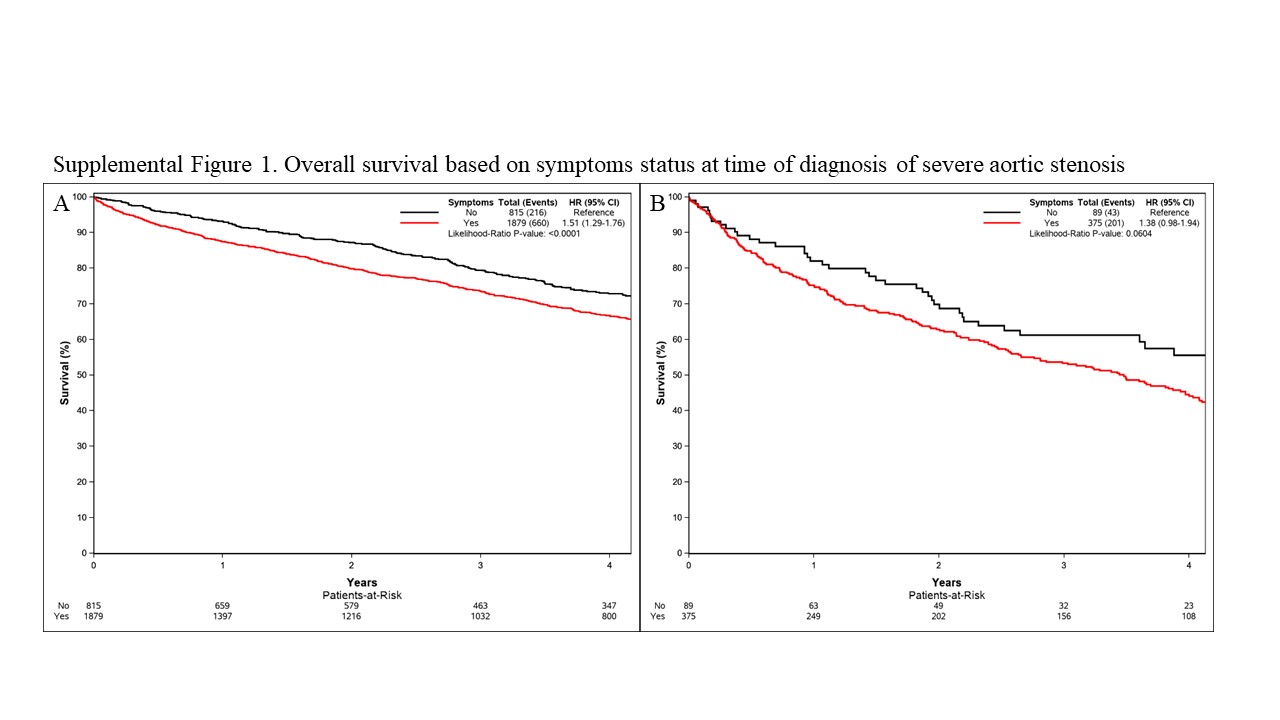

Supplement: Supplementary Figure 1 — Overall survival based on symptoms status at time of diagnosis of severe aortic stenosis. Kaplan-Meier curves for overall survival in each group based on baseline symptoms status. Survival was worse in the symptomatic versus asymptomatic group, irrespective of rhythm. Normal sinus rhythm (Panel A; p < 0.001) and atrial fibrillation (Panel B; p = 0.06). Adjusted for age, sex, and Charlson Comorbidity Index. Abbreviations: AF, atrial fibrillation; SR, normal sinus rhythm. [file Image1.jpeg]

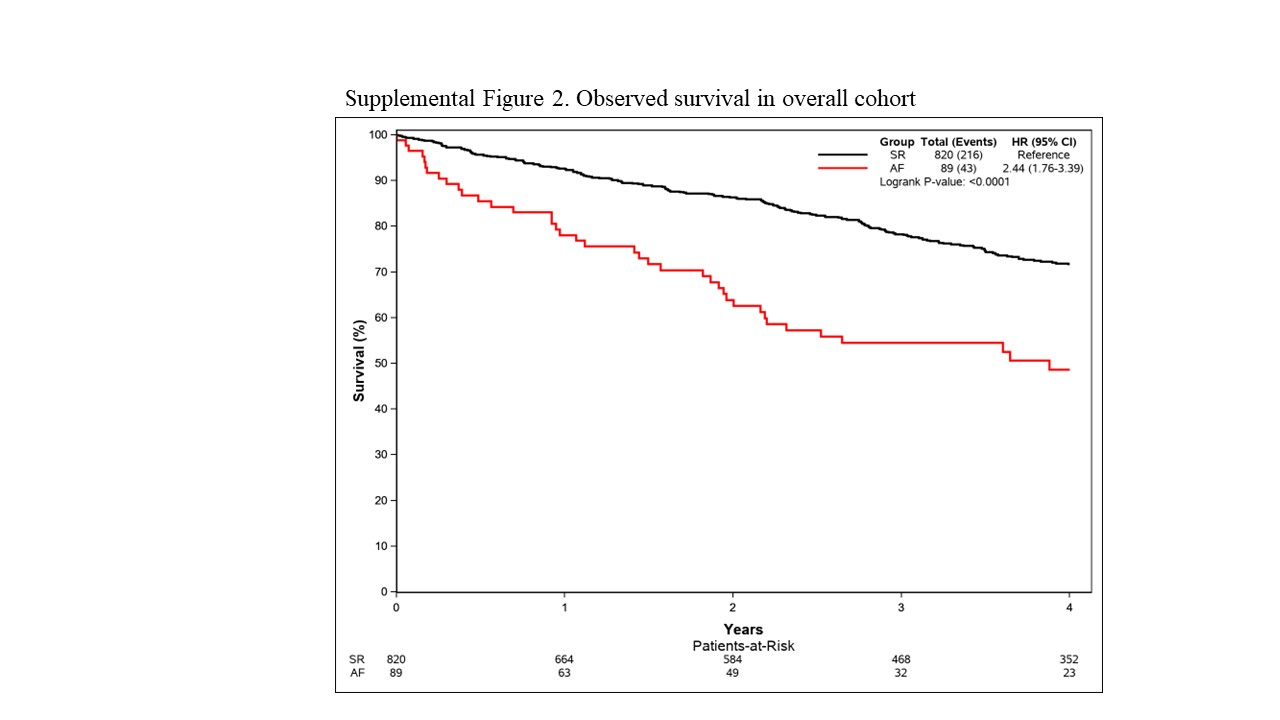

Supplement: Supplementary Figure 2 — Observed survival in overall cohort. Kaplan-Meier curves for overall survival in the overall cohort based on rhythm. Survival was worse for patients in AF versus SR (p < 0.001). Abbreviations: AF, atrial fibrillation; SR, normal sinus rhythm. [file Image2.jpeg]

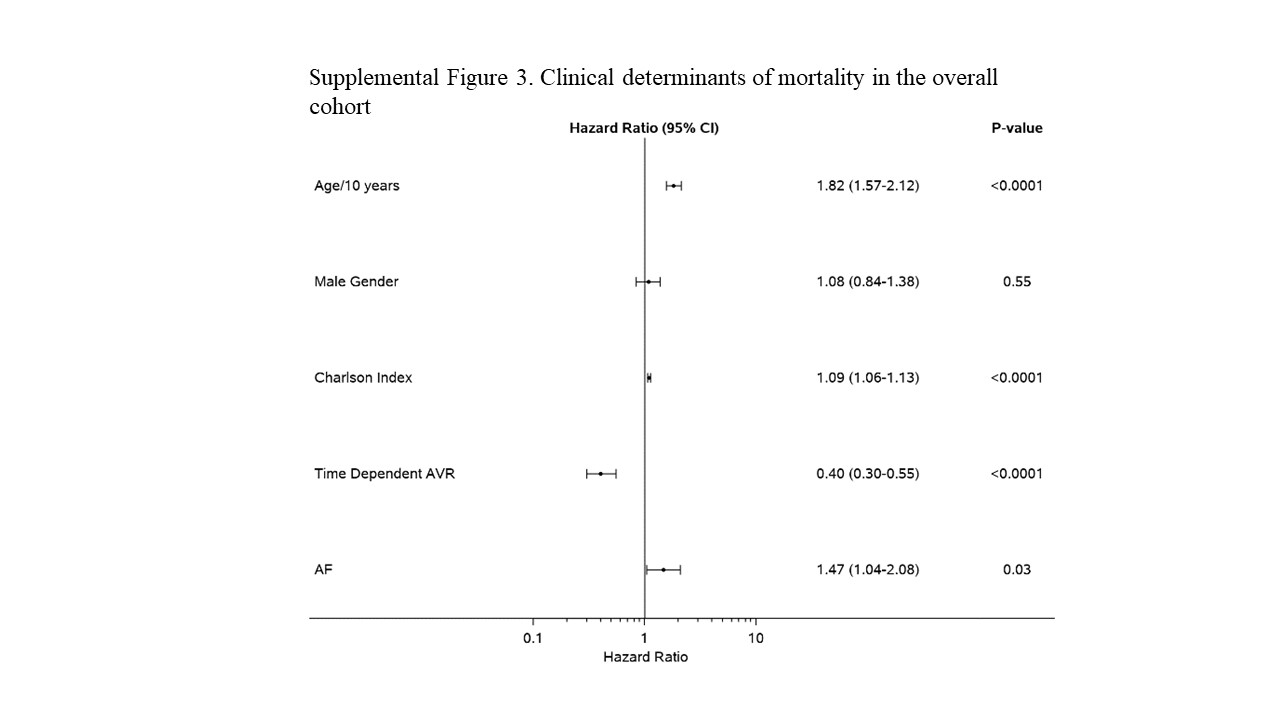

Supplement: Supplementary Figure 3 — Clinical determinants of mortality in the overall cohort. Forest plot of multivariable clinical predictors of mortality. Hazard ratios, 95% confidence intervals, and p-values from multivariable analyses are shown. Abbreviations: AVR, aortic valve replacement; AF, atrial fibrillation. [file Image3.jpeg]

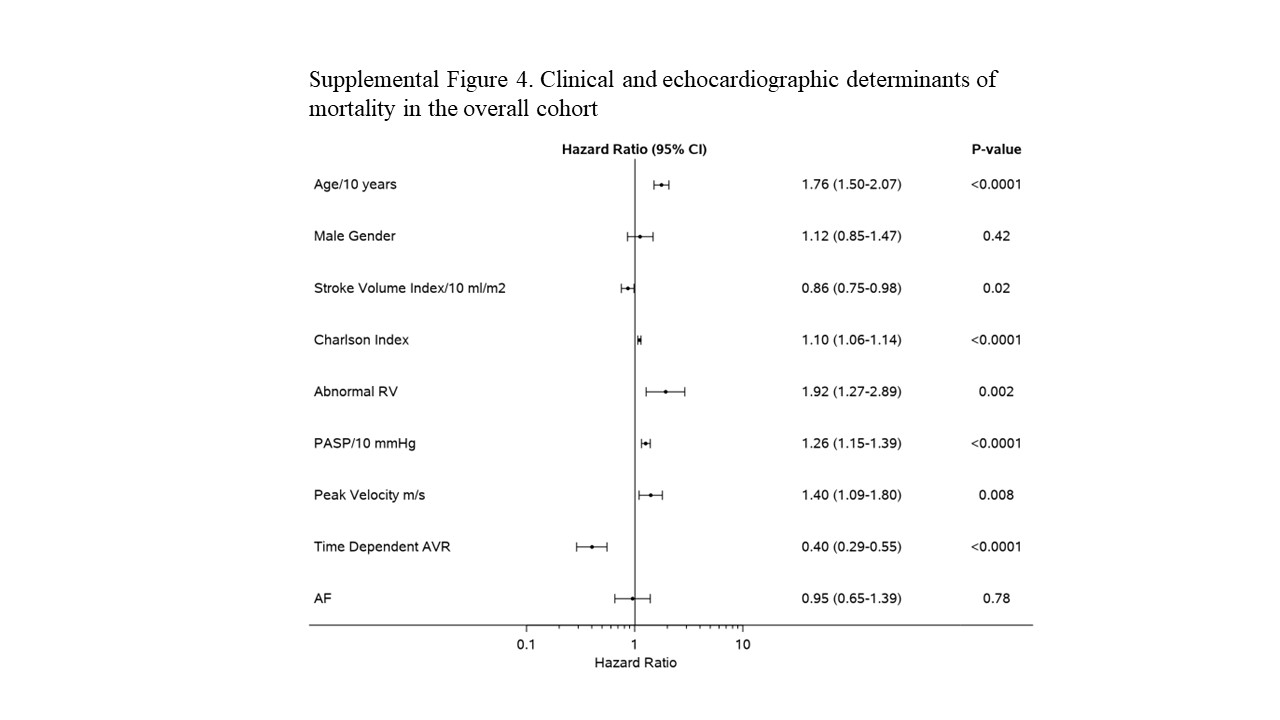

Supplement: Supplementary Figure 4 — Clinical and echocardiographic determinants of mortality in the overall cohort. Forest plot of multivariable clinical predictors of mortality. Hazard ratios, 95% confidence intervals, and p-values from multivariable analyses are shown. Abbreviations: RV, right ventricle; PASP, pulmonary artery systolic pressure; AVR, aortic valve replacement; AF, atrial fibrillation. [file Image4.jpeg]
