# Supplementary material for: Impact of atrial fibrillation on outcomes in asymptomatic severe aortic stenosis: a propensity-matched analysis
Source: Front Cardiovasc Med. 2023 Jun 20;10:1195123. doi: 10.3389/fcvm.2023.1195123 (PMC10318187; doi:10.3389/fcvm.2023.1195123)
Supplement: Supplementary file 5 [file Table1.docx]

| **Supplemental Table 1. Characteristics of Overall Cohort of Asymptomatic Patients with Severe Aortic Stenosis** | | | | | |
| --- | --- | --- | --- | --- | --- |
| **Variable** | **SR (N=820)** | | **AF (N=89)** | | ***P* Value** |
| Age | 75 | ± 11 | 82 | ± 8 | <.001 |
| Male gender, n (%) | 434 | (53%) | 52 | (58%) | 0.32 |
| Body mass index | 28.3 | ± 5.9 | 27.9 | ± 5.3 | 0.48 |
| Hypertension, n (%) | 519 | (63%) | 64 | (72%) | 0.11 |
| Diabetes mellitus, n (%) | 214 | (26%) | 30 | (34%) | 0.12 |
| Congestive heart failure, n (%) | 102 | (12%) | 34 | (38%) | <.001 |
| Chronic obstructive pulmonary disease, n (%) | 53 | (6%) | 8 | (9%) | 0.37 |
| Stroke, n (%) | 196 | (24%) | 26 | (29%) | 0.27 |
| Renal failure, n (%) | 107 | (13%) | 25 | (28%) | <.001 |
| Hyperlipidemia, n (%) | 502 | (62%) | 60 | (67%) | 0.28 |
| Coronary artery disease, n (%) | 374 | (46%) | 50 | (56%) | 0.06 |
| PCI history, n (%) | 61 | (7%) | 10 | (11%) | 0.21 |
| CABG history, n (%) | 99 | (12%) | 15 | (17%) | 0.20 |
| Charlson Index, median (Q1, Q3) | 2.0 | (1.0, 4.0) | 4.0 | (2.0, 7.0) | <.001 |
| Creatinine, median (Q1, Q3) | 1.0 | (0.8, 1.2) | 1.1 | (0.9, 1.3) | 0.012 |
| Hemoglobin, median (Q1, Q3) | 13.2 | (11.8, 14.3) | 12.6 | (11.3, 13.9) | 0.031 |
| NT-ProBNP, median (Q1, Q3) | 393 | (174, 1020) | 1916 | (1029, 3117) | <.001 |
| Ejection fraction, % | 65 | ± 6 | 62 | ± 6 | <.001 |
| Mean gradient, mmHg | 46 | ± 13 | 40 | ± 15 | <.001 |
| Peak velocity, m/s | 4.3 | ± 0.6 | 4.0 | ± 0.7 | <.001 |
| Aortic valve area, cm^2^ | 0.9 | ± 0.1 | 0.8 | ± 0.1 | 0.22 |
| Aortic valve area index, cm^2^/m^2^ | 0.6 | ± 0.1 | 0.5 | ± 0.1 | 0.09 |
| LV end diastolic dimension, mm | 48 | ± 5 | 47 | ± 5 | 0.38 |
| LV end systolic dimension, mm | 29 | ± 4 | 30 | ± 5 | 0.054 |
| Stroke volume index, mL/m^2^ | 54 | ± 12 | 43 | ± 10 | <.001 |
| Low-gradient AS, n (%) | 193 | (24%) | 39 | (44%) | <.001 |
| LV mass index, g/m^2^ | 110 | ± 27 | 107 | ± 29 | 0.36 |
| Right ventricular systolic pressure, mmHg | 35 | ± 11 | 45 | ± 14 | <.001 |
| TAPSE, mm | 22 | ± 5 | 16 | ± 4 | <.001 |
| Left atrial volume index, mL/m^2^ | 40 | ± 12 | 59 | ± 15 | <.001 |
| E/e' | 16 | ± 7 | 20 | ± 10 | <.001 |
| Q, mL/s | 265 | ± 40 | 242 | ± 44 | <.001 |
| Abnormal right ventricle, n (%) | 35 | (4%) | 22 | (26%) | <.001 |
| Mitral regurgitation^1^, n (%) | 14 | (2%) | 6 | (7%) | 0.001 |
| Tricuspid regurgitation^1^, n (%) | 18 | (2%) | 19 | (22%) | <.001 |
| Mitral or tricuspid regurgitation^1^, n (%) | 29 | (4%) | 21 | (25%) | <.001 |

SR, normal sinus rhythm; AF atrial fibrillation; PCI, percutaneous coronary intervention; CABG, coronary artery bypass graft; Q, trans-aortic flow rate; TAPSE, tricuspid annular plane systolic excursion; E, early mitral inflow Doppler velocity; e’, mitral annulus tissue Doppler velocity. ^1^Moderate or greater seve
